# Flexible e-learning video approach to improve fundus examination skills for medical students: a mixed-methods study

**DOI:** 10.1186/s12909-021-02857-8

**Published:** 2021-08-13

**Authors:** Kiyoshi Shikino, Claudia A. Rosu, Daiki Yokokawa, Shingo Suzuki, Yusuke Hirota, Katsumi Nishiya, Masatomi Ikusaka

**Affiliations:** 1 Department of General Medicine, Chiba University Hospital, Chiba, Japan; 2 Health Professional Development Center, Chiba University Hospital, Chiba, Japan; 3 Health Professions Education Program, MGH Institute of Health Professions, Boston, MA USA; 4 Department of Internal Medicine, Chiba Medical Center, Chiba, Japan; 5 Department of Diabetes, Endocrinology and Metabolism, Tokyo Medical University Hachioji Medical Center, Tokyo, Japan; 6 Center for Medical Education, Kansai Medical University, Hirakata, Japan

**Keywords:** Bloom’s taxonomy, E-learning video, Flexibility, Funduscopic, Ophthalmoscopic, Physical examination

## Abstract

**Background:**

Training for the fundus examination using traditional teaching is challenging, resulting in low generalist physicians’ confidence in performing the funduscopic examination. There is growing evidence suggesting a flexible e-learning video approach’s value in teaching physical examination procedures. However, whether the flexible e-learning video approach is superior to the traditional, face-to-face (F2F) lecture-based teaching for the funduscopic exam and the cognitive processes supporting its effectiveness has not yet been determined.

**Methods:**

We conducted a sequential explanatory mixed-method study to compare the flexible e-learning video approach’s effectiveness versus the F2F lecture-based approach for teaching the funduscopic exam to medical students at Chiba University in Japan. Medical students were randomly assigned to either a flexible e-learning video approach group or a F2F lecture approach group. We then quantitatively measured the diagnostic accuracy of funduscopic findings before and after attending the specific classrooms. Next, we conducted student focus groups to explore the students’ thinking processes in the flexible e-learning video approach vs. the F2F lecture-based teaching of fundus examination. The qualitative data were analyzed using the qualitative content analysis method.

**Results:**

The mean diagnostic accuracy scores in the post-test significantly increased from pre-test in the intervention group (36.6 to 63.4%, *p* < 0.001). Post-post comparisons across the two groups revealed a significant difference (intervention group 63.4% vs. control group 34.6%, p < 0.001). Six semi-structured focused group interviews were conducted (*n* = 36). In the flexible e-learning video approach group, we identified ten categories corresponding to four levels of the revised Bloom’s taxonomy: remember, understand, apply, analyze. Five categories were identified in the traditional F2F lecture approach group corresponding to three revised Bloom’s taxonomy levels: understand, apply, analyze. Interrater reliability was substantial (Cohen’s kappa = 0.81).

**Conclusions:**

Teaching medical students funduscopic examination using the flexible e-learning video approach leads to improved diagnostic accuracy of funduscopic examinations. The flexible e-learning video teaching method enabled higher cognitive activity levels than the traditional, lecture-based classroom, as assessed using the revised Bloom’s taxonomy.

**Trial registration:**

This study was registered with the University Hospital Medical Information Network Clinical Trials Registry on 08/02/2020 (Unique trial number: UMIN 000039434).

**Supplementary Information:**

The online version contains supplementary material available at 10.1186/s12909-021-02857-8.

## Introduction

All generalist physicians must be proficient in the funduscopic examination for detecting prevalent eye diseases such as diabetic retinopathy, hypertensive retinopathy, papilledema, and retinal hemorrhage [1, 2]. The funduscopic examination is taught to medical students and medical interns, who are expected to obtain the skills necessary to perform it [3]. However, previously published literature shows that training for the fundus examination using traditional teaching methods – where typically, the teacher is the central focus of lectures, and the primary dissemination of knowledge takes place by attending the lectures – is difficult. This results in reduced confidence on the part of generalist physicians in performing this examination [4–11]. In Japan, only 80% of medical interns and attending physicians conducted fundoscopic exams every 2–3 months, even for patients who needed them more often, according to the current guidelines [7].

A possible solution for this situation could be applying novel methods to teach the funduscopic exam, such as the e-learning video-based education. The e-learning video-based education can be an effective education method, especially in learning procedural skills [12]. Well-designed and high-quality e-learning videos may assist in filling up the gap in procedural skill training [13]. E-learning has some impact on enhancing learning because of the nature of its flexibility [14]. There is some evidence suggesting the use of a flexible e-learning video in teaching physical examination procedures [12, 13]. However, little is known about a flexible e-learning video’s usefulness in teaching funduscopic examination to medical students.

The purpose of this mixed-methods sequential explanatory study was to compare the effectiveness of the flexible e-learning video approach versus the traditional lecture-based one for teaching the funduscopic exam to medical students at the Chiba University in Chiba, Japan, by assessing the diagnostic accuracy of funduscopic findings (i.e., normal fundus, optic disc edema, pathological optic disc cupping, or not observed) for students in the flexible e-learning video approach vs. the students in the traditional, face-to-face (F2F) lecture-based approach. Furthermore, we assessed perceived changes in students’ confidence and motivation to perform the funduscopic examination via a self-administered questionnaire. We followed these with a qualitative content analysis of data obtained from focus groups conducted with a selected sample of the students participating in the study’s quantitative arm to help explain and elaborate on the quantitative results.

## Methods

### Study design overview

Using a pragmatic approach, we employed a mixed-method design that incorporated quantitative (questionnaires) and qualitative (focus groups) techniques [15–17]. This type of research study design capitalizes on quantitative and qualitative designs’ strengths while minimizing each methodology’s shortcomings. Furthermore, it allows the researchers to understand the experimental results better while incorporating the participants’ perspectives. The National Institutes of Health advises a mixed-method approach to research “to improve the quality and scientific power of data” and to better address the complexity of issues facing the health sciences today, including the health professions education [18, 19]. This study’s initial quantitative arm included a randomized controlled trial to test the teaching method’s effect on students’ skill acquisition, confidence, and motivation in performing the fundoscopic exam. The qualitative data – consisting of medical students’ perceptions – were collected after the preliminary didactics experiment. We assumed that quantitative research alone could not sufficiently capture the study participants’ cognitive processes, which seems to influence the flexible e-learning video approach’s effectiveness for improving learning. We compared the revised Bloom’s taxonomy levels of knowledge attained by the two groups of students using the qualitative data.

### Subjects and context

A randomized controlled trial design was used to compare the effects of the two methods (flexible e-learning video method vs. F2F lecture-based method) on student performance on the fundoscopic examination. The study population consisted of 104 5th year medical students at Chiba University (out of 120 total students in the 5th year) participating in a general medicine clerkship rotation from 2018 to 2019. The medical students are high school graduates (12 years of secondary school), and the medical doctor (MD) degree at Chiba University is a graduate degree that requires six years of studies. All 104 medical students signed the informed consent before enrolling in our study. Participation was voluntary and did not impact the students’ academic standing in any way. The remainder 16 medical students participated in the pilot study of this research.

None of the participants had a prior clinical clerkship rotation in ophthalmology. Participants were randomly assigned to either the flexible e-learning video groups (intervention group: *n* = 51) or the traditional F2F lecture groups (control group: *n* = 53).

### Procedure

**Fig. 1:**
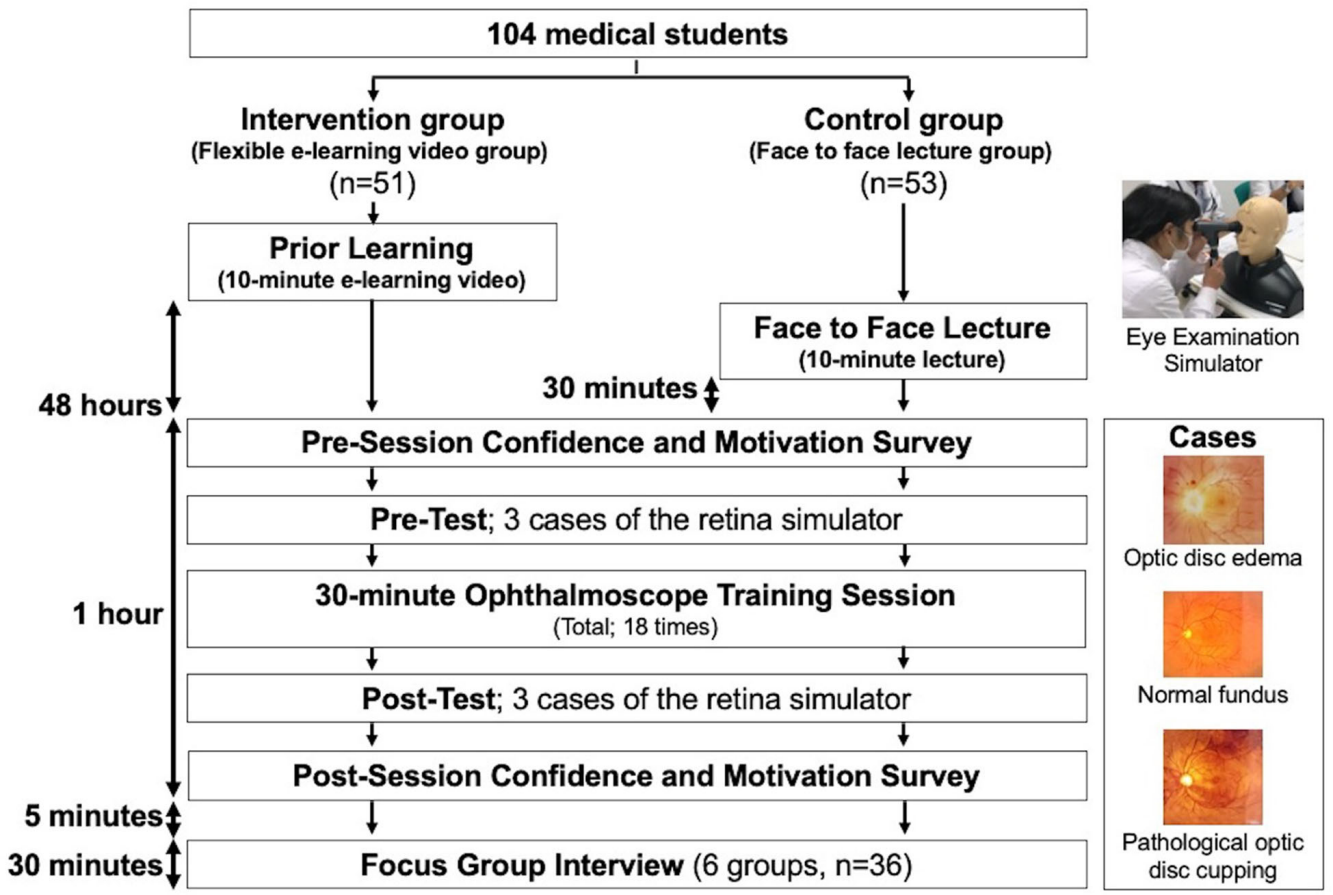
The Research Study Flow

The outline of the study design is presented in Fig. 1.

The students in the flexible e-learning video groups watched a 10-min e-learning video on fundus examination skills via a video streaming server. The video consisted of a demonstration of a person using the equipment. The video was available for viewing 48 h before completing the paper questionnaire and the pre-test simulation. They were also able to watch the video repeatedly on their smartphone, tablet, or PC. The intervention group students signed a contract not to share the video with the control group students. The traditional teaching group students also were given access to the e-learning video only after the study ended to prevent the diffusion of treatment – students in the e-learning group sharing the video with those in the lecture group. Students in the traditional teaching group attended a 10-min face-to-face lecture with similar content as the video 30 min before the retina simulator’s pre-test. The format of the lecture was the same as the video. The same images were used in both the e-learning video and lecture. Then, the faculty instructed each group about their grip, posture, procedure, angle, and light intensity. They taught fundus examination skills using the iExaminer system while sharing the iExaminer system’s images with students.

Next, students in both groups completed a paper-and-pencil questionnaire asking them about their confidence in performing funduscopic examinations and their motivation for performing funduscopic examinations. Before starting the 30-min ophthalmoscopic training session, all participants examined the eye fundus on a simulator (EYE Examination Simulator®; Kyoto Kagaku Co, Kyoto, Japan) using a PanOptic ophthalmoscope (pre-test). Each participant was assigned three cases and observed one eye for 90 s maximum per case. Participants presented their findings for each case after observing all three cases.

The iExaminer system consists of three core components: the PanOptic ophthalmoscope, the iExaminer adapter, and the iExaminer application [20]. The PanOptic ophthalmoscope addresses the fundamental challenge in ophthalmoscopy – to get a good view of the fundus to make a sufficient assessment. Patented Axial PointSource™ Optics makes it easy to enter undilated pupils, offering a 25° field of view, resulting in a view of the fundus that is five times greater than what is seen with a standard ophthalmoscope in an undilated eye. Direct viewing of the fundus through the PanOptic provides better images of the retinal changes caused by hypertension, diabetic retinopathy, glaucoma, and papilledema to enable clinicians to make these diagnoses earlier. The iExaminer adapter is designed to attach the PanOptic ophthalmoscope to the iPhone, and the iExaminer app gives the users the ability to take, store, retrieve and send fundus images right on their iPhone. It also allows medical students and their teachers to share the same visual perspective. Ophthalmoscope training session time was standardized to 30 min.

Five to six students per group participated in each ophthalmoscope training session. A total of 18 ophthalmoscope training sessions were conducted (9 in the intervention group and 9 in the control group). In each session, all the participating students were from the same group (flexible e-learning video approach or traditional F2F lecture-based approach). One of three teachers (SS, YH, and YY) was randomly assigned to each session. The teachers were previously trained using the same instructional guide (developed by KS, SS, and YH) to minimize the individual variability in guiding their students. Three iExaminer systems were available in each ophthalmoscope training session. Students performed fundus examinations on each other during the ophthalmoscope training session using the iExaminer system. They were not observed in the simulator during the ophthalmoscope training sessions.

After completing their specific training sessions, all the study participants examined the eye fundus again (post-test) on the EYE Examination Simulator® using a PanOptic ophthalmoscope. Each participant was assigned 3 cases and observed one eye for 90 s maximum per case. They presented their findings after that. Next, they completed a paper-and-pencil questionnaire asking exactly the same questions as before about their confidence in performing funduscopic examinations and their motivation for performing funduscopic examinations.

Focus groups were conducted with selected students from the two groups following the study’s quantitative arm (see “Focus Group” below for further information). After verifying that the data across the 36 selected participants possessed no excess or deficiencies in interpretation, we determined theoretical saturation and created categories, subcategories, and storylines.

This study followed the Consolidated Standards of Reporting Trials (CONSORT) reporting guideline, and the flow diagram is available (Supplement 1).

### Main outcome measures

The diagnostic accuracy of funduscopic findings (i.e., normal fundus, optic disc edema, pathological optic disc cupping, or not observed) was assessed before the ophthalmoscopic training session (pre-test) and after (post-test).

### Secondary outcome measures

An anonymous, self-administered paper-and-pencil questionnaire was employed to assess study participants’ confidence in performing funduscopic examinations and their motivation for performing funduscopic examinations before and after their respective training sessions. Each question was answered on a five-point Likert scale (from 1 - Strongly disagree to 5 - Strongly agree). KS and DY ran focus group discussions based on prior published research [21] to develop the questionnaire form.

### Statistical analysis

We performed descriptive and bivariate analyses to describe our sample. The diagnostic accuracy in funduscopic findings and the confidence and motivation for performing funduscopic examination between the groups were compared using paired t-test. Unpaired t-test was used for post-post comparisons across the group. A power analysis using the G*power computer program [22] indicated that a sample of 52 people for each group would be needed to detect small effects (f = 0.25) with 80% power and alpha set at .05. All statistical analyses were performed using IBM SPSS version 26.0 (IBM Corp. Armonk, NY).

### Focus group

A qualitative inquiry was conducted following the quantitative study to help explain the quantitative results. A sample of 36 medical students was randomly selected from the quantitative study participants (18 from the flexible e-learning video group and 18 from the F2F lecture group) [23]. After obtaining informed consent, we conducted six focus groups lasting about 30 min to minimize participants’ fatigue and regular workflow disruptions. Groups were organized separately with the students who participated in the flexible e-learning video approach (3 groups, *n* = 18) and those who participated in the F2F lecture (3 groups, n = 18).

Trained moderators (KS and DY) asked open-ended questions about students’ perception of flexible e-learning video approach effectiveness and F2F lecture teaching of fundus examination (diagnostic accuracy and the time taken to identify funduscopic findings). They asked about what went well and what did not go well in the educational session and the flexible e-learning video approach (Supplement 2). One facilitator per focus group led either an e-learning video group or a lecture group. The focus groups were recorded and transcribed verbatim. The transcripts were analyzed using deductive content analysis, drawing upon the revised Bloom’s taxonomy as the coding frame, with the cognitive process dimensions as the theme categories and subcategories [24, 25]. KS and DY did open coding of the focus group transcripts. They independently read and coded all transcripts. Then, they discussed, identified, and agreed on the coding of the descriptors. The inter-rater degree of agreement between the two researchers was assessed using Cohen’s kappa statistics.

KY derived theme categories and subcategories as they emerged from the data. The theme/categories and subcategories were regularly discussed with and reviewed for content by DY, who has extensive experience in qualitative research to ensure credibility of the findings [26]. Concepts for each of the cognitive process dimensions in the revised Bloom’s taxonomy [27] were analyzed, and the number of units of analysis for each concept was counted. The researchers have grouped similar codes into a theme and then checked to see which dimension of the cognitive process it corresponded to.

## Results

All 104 students completed the quantitative arm of this study. The participants’ mean age was 23.4 years (± 1.8), and 75.0% were men. We found no statistically significant differences in demographics between the flexible e-learning video and F2F lecture groups.

The present study focused on the differences in diagnostic accuracy of funduscopic examination, confidence, and motivation for performing funduscopic examinations between medical students enrolled in a flexible e-learning video approach vs. those enrolled in a traditional, F2F lecture-based approach.

The mean diagnostic accuracy scores in the post-test significantly increased from the pre-test in the intervention group (Table 1). Post-post comparisons across the two groups revealed a significant difference (intervention group 63.4% vs. control group 34.6%, *p* < 0.001).

**Table 1:** Diagnostic accuracy in fundus examination

		Pre-test Mean	Post-test Mean	*P*-value
**Diagnostic accuracy, % (n)**	Intervention group	36.6 (56/153)	63.4 (97/153)	< 0.001
	Control group	28.3 (45/159)	34.6 (55/159)	0.123

Intervention group; *n* = 51

Control group; *n* = 53

Confidence of funduscopic examinations and motivation to learn funduscopic examinations significantly increased from pre-test to post-test in both the flexible e-learning video and traditional F2F lecture groups (Table 2). The confidence score increased from 2.3 ± 1.3 pre-tests to 3.8 ± 0.9 post-test in the flexible e-learning video group, whereas the score increased from 2.0 ± 0.8 pre-tests to 2.8 ± 1.0 post-test in the F2F lecture group. Post-post comparisons across the two groups revealed a significant difference (intervention group 3.8 vs. control group 2.8, *p* < 0.001). The motivation score increase in the post-test vs. pre-test was 3.5 ± 0.7 to 4.4 ± 0.6 in the flexible e-learning video group and 3.3 ± 1.0 to 3.9 ± 0.9 in the F2F lecture group. Post-post comparisons across the two groups revealed a significant difference (intervention group 4.4 vs. control group 3.9, p < 0.001).

**Table 2:** Confidence of funduscopic examinations and motivation to learn funduscopic examination

		Pre-teaching Mean	Post- teaching Mean	*P*-value
**Confidence of funduscopic examinations**^a^**(SD)**	Intervention group	2.3 (1.3)	3.8 (0.9)	< 0.001
	Control group	2.0 (0.8)	2.8 (1.0)	< 0.001
**Motivation to learn funduscopic examinations**^a^**(SD)**	Intervention group	3.5 (0.7)	4.4 (0.6)	< 0.001
	Control group	3.3 (1.0)	3.9 (0.9)	0.002

Intervention group; *n* = 51

Control group; *n* = 53

^a^1: Strongly disagree, 5: Strongly agree

### Content analysis

The educational method’s (flexible e-learning video approach vs. traditional lecture-based approach) effect on the students’ cognitive processes between intervention and control groups was explored. Our analysis categories were preset according to the six cognitive process levels from the revised Bloom’s taxonomy. Following open coding, similar codes were grouped into a theme. Then, each theme was checked against the revised Bloom’s taxonomy’s definitions to see which level of the cognitive process it corresponded. Thematic saturation was reached after analyzing transcripts from 6 focus groups, three each from the intervention and control groups. The absolute frequencies of the codes for each cognitive process dimension for our data are presented in Tables 3 and 4. In the intervention group, a total of ten categories and eighteen subcategories were identified corresponding to four levels of the revised Bloom’s taxonomy [28]: remember, understand, apply, analyze (Table 3). A total of five categories and six subcategories were identified in the control group corresponding to three levels of the revised Bloom’s taxonomy: understand, apply, analyze (Table 4). The most frequent subcategory by the number of codes was “Analyze” in the flexible e-learning video group and “Apply” in the F2F lecture group. The interrater reliability was substantial (Cohen’s kappa = 0.81).

**Table 3:** Absolute frequencies of codes for each theme (Flexible e-learning video classroom group)

Flipped classroom approach				
Theme (Cognitive process dimension)	Category	Subcategory	Number of codes	Quotes
Analyze	Effective learning	Efficient acquisition of fundus examination skill	6	“I don't even know what I'm not doing when I don't know anything. I realized that because I prepped.”
		Effectiveness of preparation	4	
		Establishing by repetition	1	
		Linking knowledge and practice	1	
	Analysing procedure	Analysis of fundus examination performance	3	
	Psychological safety	Feeling safe for practicing	2	
		Anxiety about preparation	3	
Apply	Learning motivation	Motivation for performing real patient	3	“I was even able to produce a field of view, which I would like to do if I have the opportunity in the future.”
		Motivation from successful experiences	1	
	Using abstractions	Application of knowledge obtained in preparation	1	
		Practice using the key points of fundus examination	1	
	Concrete experience	Imaging of diagnostic procedures	1	
	Problem solving	Trouble shooting of equipment	1	
Understand	Understanding procedure	Observation method of fundus	9	“I was able to understand basic things such as looking from 15 degrees outward from the video without the teacher’s advice.”
		Usage instructions of fundoscope	1	
	Efficient understanding	Efficient understanding of fundus examination	2	
Remember	Memory retention	Improve memory retention through preparation	6	“I felt that the connection with the actual experience really helped me to retain the memory.”
	Acquisition of knowledge	Acquisition of necessary knowledge for fundus examination	4

**Table 4:** Absolute frequencies of codes for each theme (Traditional F2F lecture-based approach group)

Traditional approach				
Theme (Cognitive process dimension)	Category	Subcategory	Number of codes	Quotes
Analyze	Analysing procedure	Analysis of fundus examination performance	1	“I thought that was great fun because it was easy to see when I actually put that into practice.”
Apply	Concrete experience	Practice of fundus observation	7	“I had never been able to see the fundus in person before, but this lecture was the first time I was able to see it.”
	Learning motivation	Motivation from successful experiences	1	
	Using abstractions	Application of knowledge obtained in lecture	1	
Understand	Understanding procedure	Observation method of fundus	2	“At least I now know how to use a fundoscope.”
		Usage instructions of fundoscope	2	

## Discussion

This study suggests that the flexible e-learning video teaching method is superior to the traditional F2F lecture-based teaching method for fundus examination training among medical students. Our results showed that the flexible e-learning video students significantly improved their diagnostic accuracy of fundus examination compared to the traditional F2F lecture-based teaching group. Additionally, the content analysis results showed that the flexible e-learning video group achieved Bloom’s taxonomy levels “Analyze,” “Apply,” “Understand,” and “Remember.” The F2F lecture group achieved levels of “Analyze,” “Apply,” and “Understand” of the same taxonomy. Furthermore, while the intervention group expressed mainly the subcategories and codes of the “Analyze” level in Bloom’s taxonomy, the F2F lecture group concentrated mainly on the “Apply” level. This may be because the flexible e-learning video teaching method may enable higher levels of cognitive activity according to the revised Bloom’s taxonomy [27–29].

The flexible e-learning video teaching method may offer advantages for ophthalmology medical education for teaching the basic knowledge of fundus examination. The knowledge required for fundus examination can be acquired during early medical education stages, e.g., medical school [30] using the flexible e-learning video teaching approach. The e-learning video may also assist in filling up a gap in procedural skill training [13]. The present study results suggest that fundus examination skills were improved through high-dimensional learning and reflection such as application and analysis enabled by having the foundational knowledge through prior viewing of an e-learning video.

This study also showed that the flexible e-learning video group students significantly increased their confidence in and motivation for performing fundus examinations compared to the F2F lecture-based teaching group. Low confidence levels of medical interns and attending physicians in their fundoscopic examination skills were linked to decreased rates of providing this exam [9–11]. One challenge for teaching the fundus examination is that medical students and physicians alike have reduced confidence using an ophthalmoscope [31, 32]. As such, few clinicians perform ophthalmoscopy, and many who do are unable to detect abnormalities of the ocular fundus reliably. Our study indicates that the flexible e-learning video approach improves medical students’ confidence in fundus examination; thus, it is expected that the frequency of funduscopic examination in clinical settings will increase.

Furthermore, the flexible e-learning video teaching method increased students’ motivation to learn fundus examinations. In light of psychological findings, it has been consistently demonstrated that learning motivation predicts learning outcomes, and learning motivation is an essential foundation for knowledge and skill acquisition, thinking, and expression [33]. Thus, teaching the fundus examination using the flexible e-learning video method can motivate medical students to continue to perform fundus examinations.

Several factors are thought to impact the results of this study [34–36]:

*Student factors*: preference for and experience with video-based learning, desire for flexibility in timing, and ability to view lectures in a distraction-free environment, increasing their comprehension.

*Instructor factors*: live lectures have variation in quality, inability to pause/rewind/re-watch may limit uptake of some critical information, interpersonal/relational dynamics between instructor and students may impact learning.

*Content/modality factors*: the fundoscopic images in the presentation may be better viewed on a computer screen rather than on a projector screen. A complex terminology may be easier to hear in a recording with headphones than in a large classroom with other auditory distractions. Additionally, the small instruments may be easier to explain on a large image on a computer screen rather than a live demonstration with some distance between the instructor and students.

### Limitations

This study has several limitations. We did not measure how many times the e-learning videos were viewed or how long the video was played. Thus, the intervention group students may have had more exposure/“time on task” than the control group students, who only had one 10-min lecture.

The present research was conducted with a simulator, not with actual patients. Although our intervention was effective in the simulation settings, it is necessary to verify whether it can be used, with the same effects, with real patients. Third, there is a possibility that the educational effect of the flexible e-learning video teaching for fundus examination depends on the teaching skills of the faculty. However, in our study, we designed the faculty’s instruction and training to minimize the effects of faculty teaching skills. Furthermore, this study was conducted in classrooms where the faculty deliberately chose to experiment with the flexible e-learning video approach to teaching fundus examination; thus, our results may not be generalizable or transferrable beyond the specific population from which the sample was drawn. Additional validation is needed to determine the applicability of these results to residents and general physicians, too.

## Conclusion

Teaching funduscopic examination to medical students using the flexible e-learning video approach improves medical students’ diagnostic accuracy, confidence, and motivation to perform funduscopic examinations. The flexible e-learning video is an effective and efficient method for teaching fundoscopic examination techniques, leading to improved competency outcomes in medical students.

## Supplementary Information

**Additional file 1.** This study followed the Consolidated Standards of Reporting Trials(CONSORT).

**Additional file 2.** The study participants were asked about what went well and what did not go well in the educational session and the flexible e-learning video approach.

## Data Availability

The raw dataset supporting the conclusions of this article is available from the corresponding author upon request.
