# Supplementary material for: Flexible e-learning video approach to improve fundus examination skills for medical students: a mixed-methods study
Source: BMC Med Educ. 2021 Aug 13;21:428. doi: 10.1186/s12909-021-02857-8 (PMC8364022; doi:10.1186/s12909-021-02857-8)
Supplement: Supplementary file 1 — Additional file 1. This study followed the Consolidated Standards of Reporting Trials(CONSORT). [file 12909_2021_2857_MOESM1_ESM.docx]

**Supplement 1: Study flow diagram**


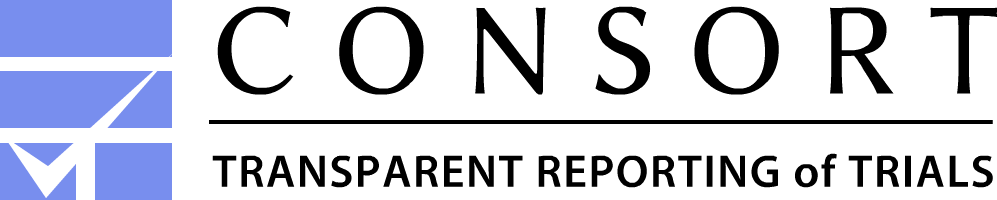


**CONSORT 2010 Flow Diagram**

Allocated to intervention (n=51)

## Follow-Up

Analysed (n=51)
♦ Excluded from analysis (n=0)

## Analysis

Analysed (n=53)
♦ Excluded from analysis (n=0)

Lost to follow-up (n=0)

Discontinued intervention (n=0)

Lost to follow-up (n=0)

Discontinued intervention (n=0)

## Enrollment

## Allocation

Allocated to control (n=53)

Randomized (n=104)

Excluded (n=0)

Assessed for eligibility (n=104)
