# Supplementary material for: Flexible e-learning video approach to improve fundus examination skills for medical students: a mixed-methods study
Source: BMC Med Educ. 2021 Aug 13;21:428. doi: 10.1186/s12909-021-02857-8 (PMC8364022; doi:10.1186/s12909-021-02857-8)
Supplement: Supplementary file 2 — Additional file 2. The study participants were asked about what went well and what did not go well in the educational session and the flexible e-learning video approach. [file 12909_2021_2857_MOESM2_ESM.docx]

**Supplement 2: Basic interview and focus group schedule**

- Can you tell me something about your past experiences doing fundoscopic examination?
- What was it like to perform the fundoscopic examination in this session?
- How do you evaluate your fundoscopic examination performance?
- At what point do you think it worked for you?
- Why do you feel this way?
- At what point do you think it did not work for you?
- Why do you feel this way?
- How has the use of flipped classroom teaching / traditional teaching impacted your fundus examination training?
- What is the significance of incorporating flipped classroom teaching / traditional teaching in this fundus examination session?
- What do you think is the significance of preparation for the session and attending the training session?
- What are some points where you were able to take advantage of preparation for the session?
